# Impact of Phlebotomy on Quality of Life in Low-Risk Polycythemia Vera

**DOI:** 10.3390/jcm13164952

**Published:** 2024-08-22

**Authors:** Nathan Visweshwar, Bradley Fletcher, Michael Jaglal, Damian A. Laber, Ankita Patel, Jennifer Eatrides, Geetha Rajasekharan Rathnakumar, Keshav Visweswaran Iyer, Irmel Ayala, Arumugam Manoharan

**Affiliations:** 1 Division of Hematology, University of South Florida, Tampa, FL 33612, USA; bsfletcher@usf.edu (B.F.); keshaviyer322@gmail.com (K.V.I.); 2 Department of Hematology and Hematological Oncology, Moffitt Cancer Center, Tampa, FL 33612, USA; michael.jaglal@moffitt.org (M.J.); damian.laber@moffitt.org (D.A.L.); ankita.patel@moffitt.org (A.P.); jennifer.eatrides@moffitt.org (J.E.); geetha.rajasekharanrathnakumar@moffitt.org (G.R.R.); 3 Division of Hematology, Johns Hopkins All Children’s Hospital, St. Petersburg, FL 33701, USA; irmel.ayala@jhmi.edu; 4 Faculty of Science, Medicine and Health, University of Wollongong, Wollongong, NSW 2522, Australia; arumugam@uow.edu.au

**Keywords:** polycythemia vera, low risk, phlebotomy, quality of life, chemotherapy

## Abstract

Polycythemia vera is an indolent myeloproliferative disorder that predisposes patients to venous and arterial thrombosis and can transform into myelofibrosis and acute myeloid leukemia. Consistent phlebotomy prevents life-threatening cerebrovascular and coronary artery disease and prolongs survival in low-risk polycythemia vera (patients under 60 years without thrombosis). However, despite its effectiveness in preventing serious complications, phlebotomy does not necessarily enhance the quality of life (QoL). This review assesses QoL issues associated with low-risk PV, explores alternative management strategies such as erythrocytapheresis, and discusses the roles of hydroxyurea, peginterferon, ruxolitinib, and other novel agents in potentially improving disease management and patient outcomes.

## 1. Introduction

Polycythemia vera (PV) is a myeloproliferative disorder that presents with leukocytosis, arterial and venous thrombosis, fatigue, pruritus, and progression to myelofibrosis. Its incidence is 2 per 100,000 [1]. Arterial thrombosis accounts for 34% of thrombotic complications, and 20–40% of these are fatal [2]. The overall survival for low-risk PV patients is approximately 15 years [3].

Polycythemia vera treatment is based on risk stratification. Low-risk patients with PV include those who are less than 60 years old without a history of thrombosis. A randomized study found that patients receiving therapeutic phlebotomy to maintain 0.45 HCT and low-dose aspirin were less likely to die from cardiovascular disease than those with 0.45 to 0.50 HCT [4].

In low-risk PV, other treatments such as chemotherapy are thought not to improve survival. However, in a comparative study, the survival resulting from phlebotomy alone was 14 years, while the cytoreductive group’s survival was 20 years. Both groups were similar in age, with median ages of 53 and 54, respectively [4]. Additionally, according to the REVEAL study, age was not associated with venous thrombosis, but leukocytosis was associated both with venous and arterial thrombosis, even in the low-risk category [5]. Besides phlebotomy, there is emerging evidence that other treatments reduce thrombotic risk. These include hydroxyurea, interferon, and ruxolitinib.

The European Leukemia Net (ELN) report suggests that low-risk PV patients with certain poor risk features (intolerance to phlebotomy, symptomatic splenomegaly, severe disease-related symptoms, platelet counts greater than 1500 × 10^9^/L or a leukocyte count higher than 15 × 10^9^/L, or inadequate HCT control with phlebotomy) may benefit more from cytoreductive treatment, JAK 2 inhibition, peginterferon, or allogenic bone marrow transplantation than phlebotomy alone [6]. Here, we discuss quality of life (QoL) issues with phlebotomy in patients with low-risk PV and review alternative strategies for their management.

## 2. Methods

### 2.1. Search Strategy

For this narrative review, we undertook a literature search in the Medline, Embase, and Cochrane databases for studies published up to February 2024. Peer-reviewed articles, clinical trials, case reports/series, and abstracts published in peer-reviewed journals were screened. The MeSH terms used included polycythemia vera; low risk; phlebotomy; hydroxyurea; interferon; JAK2; and QoL. Presentations at national and international conferences including those of the American Society of Hematology, the European Society of Hematology, and the British Society of Hematology were studied. There was a restriction on language (non-English studies were not reviewed), but there were no restrictions on patient age or ethnic group. The data for this manuscript were obtained on author names, intervention, comparison details, and outcomes from the literature search. Further references were obtained from material retrieved by the manual search and from the bibliographies of the retrieved articles.

#### 2.1.1. Ideal HCT and Difficulties in Focusing Solely on HCT in the Management of Low-Risk PV

In low-risk PV, HCT is used to guide treatment because it has been shown to reduce the risk of thrombosis [4]. In the CYTO-PV study, keeping HCT below 0.45 through phlebotomy or HU significantly decreased cardiovascular mortality and thrombosis [4]. A lower target hematocrit range of 40–42% is recommended for individuals with persistent or recurrent symptoms of hyperviscosity, such as erythromelalgia, transient ocular attacks, headaches, dizziness, and/or amaurosis fugax [7]. This adjustment is particularly important when symptoms persist despite a standard target hematocrit of 45%. The decision to aim for a lower target should be guided by documented clinical benefit and symptom relief. In low-risk PV, the most common cause of thrombosis is a failure to adhere to the phlebotomy regimen (with a dropout of about 30%) of patients who did not maintain an HCT of 0.45 or less [8]. However, it is shown that strict adherence to HCT may be associated with a higher symptom burden. These symptoms include fatigability, pruritus, lack of concentration, insomnia, weight loss, and night sweats [9].

Repetitive venesection results in decreased red cells, which take up less space, thus lowering the HCT. Despite a normal HCT, there is increased risk of thrombosis [10]. When phlebotomy numbers increase due to microcytosis and decreased MCV, the HCT is an inaccurate measure of true red blood cell counts. In the long run, phlebotomy does not seem to be an effective therapeutic method for containing red cell mass. With significant splenomegaly, the hematocrit may also be normal due to increased plasma volume in PV [11]. In low-risk PV, despite the control of HCT, persistent PV-related symptoms secondary to splenomegaly, leukocytosis, and cytoreduction lead to poor quality of life [11]. At the start of treatment, most PV patients have no iron deficiency affecting the mean corpuscular volume (MCV) of the red blood cells. So, the HCT value can guide phlebotomy at the beginning. However, when phlebotomy sessions increase to three or four per year, the HCT is an inaccurate measure of true red blood cell counts. Finally, thrombosis may occur with normal HCT in patients with low-risk PV because of leukocytosis and a JAK2-positive status [12].

#### 2.1.2. Negative Outcomes Despite Achieving Target HCT in Low-Risk PV

The results of clinical trials conducted by the PV study group challenge the existing belief that thrombosis is less common among patients who have undergone phlebotomy than among those who have received myelosuppressive therapy in the first three years of treatment in low-risk PV [13]. The thrombotic risk was not associated with poorer disease control, as indicated by HCT [14]. The PVSG 05 trial was stopped at a median follow-up of 2 years when 8.0% patients developed a major thrombosis after phlebotomy, aspirin, and dipyridamole, compared with 2.2% in the chemotherapy arm. This further demonstrates that therapeutic phlebotomy is associated with a higher rate of thrombosis than chemotherapy in low-risk PV [15]. Based on an analysis of 1042 patients with PV in the ECLAP trial, hydroxyurea therapy was found to be superior to phlebotomy with respect to fatal and nonfatal cardiovascular events: 13.2% in phlebotomy versus 7.9% in hydroxyurea treatment (*p* = 0.006) [16].

During the management of PV, phlebotomy alone is found to be ineffective, as it does not help with leukocytosis, thrombocytosis, or progression to myelofibrosis [17]. Leukocytosis is associated both with venous and arterial thrombosis even in the low-risk category [5]. Because of the lack of compliance with phlebotomy, about 47% had HCT greater than 50%, leading to increased risk of thrombotic events [18]. Studies have shown a higher rate of thrombosis in patients treated with hydroxyurea with 3 or more phlebotomies per year compared to hydroxyurea with 0–2 phlebotomies per year (20.5% vs. 5.3% at 3 years; *p* < 0.0001) [19].

Iron deficiency increases hypoxia-inducible factor (HIF), which inhibits hepcidin, a master regulator of iron metabolism, increasing erythropoiesis, red cell mass, and thrombosis [20]. There is a significant and independent association between therapeutic phlebotomy and an increased risk of thrombosis, but there was no correlation between the intensity of the phlebotomy regimen and thrombosis risk [21].

In a health care claims database study, almost half of patients with PV had inadequate hematocrit control during their lifetime, with hematocrit values intermittently exceeding 50% with increased incidence of thrombosis. In order to reduce thrombotic complications, hematocrit control must be stable, so intermittent phlebotomy’s efficacy is limited when HCT is >45% between treatments [22]. Furthermore, phlebotomy alone does not address the underlying disease process, causing pruritus and fatigue [6].

There is conflicting evidence regarding the role of therapeutic phlebotomy with leukocytosis and its part in thrombosis from retrospective studies in PV [23]. There appears to be a significant risk of thrombosis associated with leukocytosis in low-risk PV patients due to the qualitative and quantitative defects of leukocytes in PV patients [12]. The current guidelines specify that cytoreductive treatment is recommended based on leukocytes > 15 × 10^9^/L [6]. However, in a retrospective study, PV patients with constantly elevated leukocyte counts had similar rates of thrombotic events, but progressed to myelofibrosis, myelodysplastic syndrome, or AML more often than those without leukocytosis [24]. The prospective observational study (REVEAL) confirmed that leukocytosis and thrombocytosis have higher risk of arterial thrombosis, whereas female sex and a history of previous thrombosis were associated with venous thrombosis. Persistent leukocytosis is associated with thrombosis in PV patients, even when the HCT is adequately controlled [5].

Manoharan et al., in a risk-adjusted study of 132 patients with myeloproliferative disorders including PV (27), demonstrated equal efficacy of aspirin, clopidogrel (75 mg daily), and odorless garlic. In a follow-up period of 1–23 years (median 8 years), none of these patients developed thrombosis [25].

#### 2.1.3. Impaired QoL in Patients with Low-Risk PV

Polycythemia vera is associated with long-term physical and psychological complications such as fatigue, headaches, weight loss, sweating, and pruritus. Studies continue to show that PV symptoms remain undertreated despite many advancements in treatment [26]. Among 405 patients with PV receiving standard treatment, the authors found that fatigue (85%), pruritus (65%), night sweats (49%), bone pain (43%), fevers (13%), and unwanted weight loss (10%) contributed directly to poor QoL [26].

Up to 65% of the PV population are affected by pruritus, a condition characterized by generalized itching, prickling, or burning that is known to contribute to poor QoL [3]. A variety of stimuli can trigger pruritus, including hot water, physical activity, sweating, alcohol consumption, and humidity. The sensation of itching may also be accompanied by a variety of other symptoms, such as anger, hostility, aggression, embarrassment, and even suicidal thoughts [27]. Based on the EORTC QLQ30, a German study of 441 PV patients with aquagenic pruritus found that patients with pruritis had significantly worse QoL (*p* = 0.0007) than patients without pruritus [28]. The decline in QoL in low-risk PV patients after phlebotomy was associated with a reduction in cognitive (*p* = 0.0014), emotional (*p* = 0.0028), and social function (*p* of 0.00010) [29]. Polycythemia vera has similar effects on QoL as other myeloproliferative neoplasms (MPNs) and advanced cancers. According to a MPN QoL survey, 79 to 86% of PV patients reported that the disease affected their quality of life (average disease duration, 7.8–9.1 years) [30]. Patients with PV have poor QoL among all patients with myeloproliferative disorders [31]. In addition to poor QoL and frequent falls, poor exercise tolerance is also a burden to caregivers due to the constant attention required and the time spent driving patients to phlebotomy clinics [32].

Only focusing on thrombosis risk can lead to poor QoL and increased anxiety and depression for the next 15–20 years [33]. According to studies, almost all PV patients present with constitutional symptoms at diagnosis that cannot be alleviated by phlebotomy alone. In individuals presenting with symptoms potentially attributed to phlebotomy-induced severe tissue iron deficiency—such as pica, oral paresthesia, esophagitis, and restless legs—iron supplementation is advised. Addressing iron deficiency can alleviate these symptoms and improve overall well-being [7].

In low-risk polycythemia vera patients, phlebotomy can cause depression and lack of energy. There is limited evidence supporting the efficacy of stimulants, including methylphenidate and modafinil, in treating deconditioning and depression [34]. Low-risk PV patients appear to have a higher incidence of fatigue than the general population. While receiving therapy for MPN (myeloproliferative neoplasm), symptoms of insomnia, depression, and anxiety persisted in 1971 patients (34.7% with PV) [35]. A decline in QoL is relevant because PV patients are expected to live a normal lifespan, suggesting that phlebotomy may not be the most beneficial treatment in patients with low-risk PV.

#### 2.1.4. Phlebotomy, Iron Deficiency, and QoL in Low-Risk PV

Secondary to therapeutic phlebotomy, patients develop iron deficiency. Iron deficiency limits hemoglobin synthesis by suppressing erythropoiesis through the aconitase-associated regulatory pathway controlling erythroid differentiation, the blockade of lysosomal trafficking of transferrin receptor 2, and the disruption of the erythroblast cell cycle [36]. In addition to erythropoiesis, iron is essential for neurological, musculoskeletal, and psychological development. At the cellular level, phlebotomy results in iron deprivation with defective DNA synthesis with G2/M arrest, leading to poor performance [37]. As a result, patients with iron deficiency experience lethargy, impaired psychomotor development, physical disability, cognitive impairment, learning difficulties, glossitis, cheilosis, koilonychias, restless legs syndrome, cravings for ice, flour, and chalk, and poor QoL [38]. Besides anemia, iron deficiency causes fatigue due to altered mitochondrial function and myoglobin production, cytochrome dysfunction, adenosine triphosphate production dysfunction, and failure of the cell cycle to regulate itself [39]. Symptoms of iron deficiency can be confused with symptoms of PV. Phlebotomy may also have a counterproductive effect on patients with low-risk PV by increasing prothrombotic gene transcript levels [40].

#### 2.1.5. Impact of Sexual, Social, and Psychological Issues on QoL

In patients with polycythemia, depression is more common than in the general population [35]. A study of MPN patients (34% with PV) found that 64% struggled with sexuality issues, out of which 38% were severe [41]. Physical symptoms such as pruritus, fatigue, or depression associated with treatment can lead to a lack of sexual interest [35]. Among PV patients, up to 57% of both males and females suffer from debilitating sexual dysfunction [42]. There is a strong correlation between depression and sexual dysfunction in patients with MPNs, and the rate is as high as 77% (34% PV), a much higher level than in the general population [35]. Patients with MPNs, PV, and ET are likely to suffer from complaints that are heavily influenced by sexuality [43]. Sexuality-related complaints, insomnia, depression, and night sweats have all been linked to endothelial dysfunction. Psychological and biological factors appear to have a complex pathophysiology. The vascular tone of PV patients without arterial disease can be impaired in addition to arterial and venous thrombotic complications [44]. In females, fatigue is the most common symptom contributing to decreased sexual arousal and a lack of interest [45]. Abdominal discomfort is also common in females with PV, regardless of whether they have intraabdominal thrombosis.

## 3. Quality-of-Life Assessment

In 2007, based on an Internet-based symptom survey of 1179 patients with PV, essential thrombocythemia (ET), and MF, the first QoL assessment tool was designed [26]. The symptom assessment tools utilized were the Functional Assessment Cancer Therapy-Anemia (FACT-An) and the Brief Fatigue Inventory (BFI) [46,47]. In an evaluation of QoL conducted using a modified SFID questionnaire (Symptom, Frequency, Intensity, Distress), none of the parameters reported in the questionnaire were alleviated by treatment [48]. In 2012, myeloproliferative disorders, such as ET and PV, were first standardized using the Myelofibrosis Symptom Assessment Form (MF-SAF). Diminishing energy for daily routine tasks was primarily caused by fatigue. In all myeloproliferative disorders, including PV, fatigue is a major contributing symptom [49]. The symptoms assessed by the MF-SAF were night sweats, abdominal discomfort, pruritus, weight loss, fever, cough, inactivity, fatigue, bone pain, and early satiety [42]. Additionally, the list was expanded to include dizziness, lightheadedness, insomnia, sexual dysfunction, vertigo, headaches, and numbness/tingling [50]. According to the MPN Landmark survey, many respondents reported a reduced work schedule, sickness interrupting work, and terminating their jobs because of their MPN. In many cases, MPN-related symptoms negatively affected respondents’ quality of life (MF, 81%; PV, 66%;) [51].

**Figure 1:**
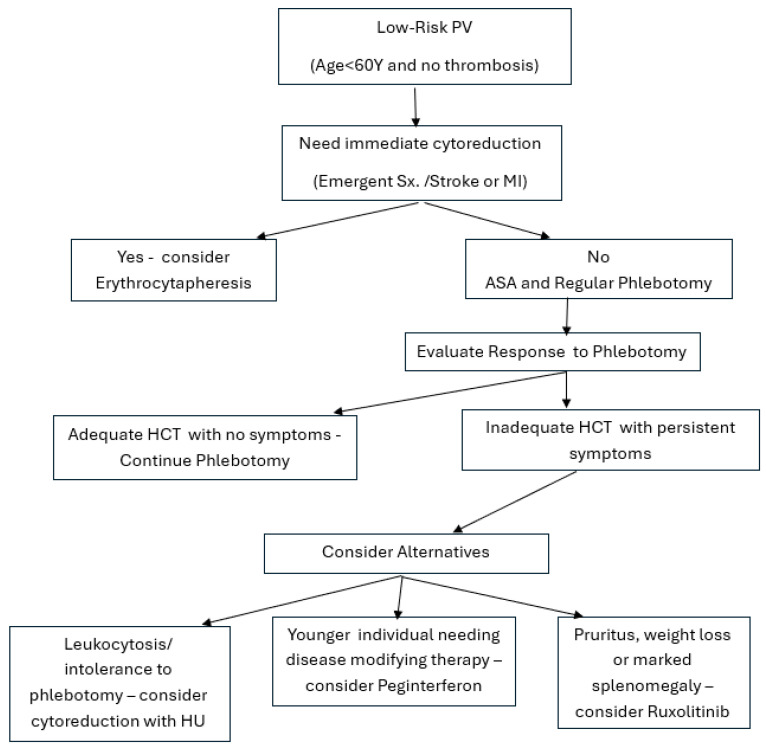
Suggested algorithm for the management of low-risk PV.

The PVSG-01 and CYTO-PV studies provided the basis for current recommendations on phlebotomy in PV [14]. In the PVSG-01 study, patients were randomly assigned to receive either phlebotomy alone or phlebotomy with P32 or chlorambucil. During the first three years, phlebotomy alone was associated with a higher risk of thrombosis, and chemo/radioisotope therapy resulted in leukemic transformation and secondary cancers. The CYTO-PV study randomly assigned patients receiving phlebotomy, HU, or both a target HCT of 0.45 or a HCT between 0.45 and 0.50. A follow-up duration of 31 months revealed 5 thrombotic episodes or cardiovascular deaths in the group with HCT < 0.45 (2.7%) and 18 deaths in the group with HCT > 0.45 (9.8%). Based on these two studies, the current guidelines and evidence-based treatment recommendations aim to reduce the risk of thrombosis and fatal cardiovascular events. Nevertheless, in low-risk PV patients, other agents are necessary because of the poor QoL from iron deficiency caused by repeated phlebotomy (Table 1). Furthermore, phlebotomy alone does not prevent progression of the disease. Novel strategies with early interventions in low-risk PV patients may improve outcomes and decrease disease progression (Figure 1). Young patients with low-risk PV with no history of prior thrombosis are usually symptomatic and are at risk of progression over the long term. There appears to be no difference in the cumulative incidence of progression to myelofibrosis (MF) between ELN/National Cancer committee network (NCCN) low-risk and high-risk patients [52].

## 4. Hydroxyurea

The role of hydroxyurea (HU) in the treatment of low-risk PV remains unclear. There is no robust evidence for HU to be used as a first-line therapy, and normalizing complete blood counts has not been found to be beneficial for reducing thrombosis risk [55]. Hydroxyurea does not prevent thrombosis or prolong survival. There was, however, significant improvement in the percentage of fatal/non-fatal CV events in a cohort of patients with PV compared to hydroxyurea or phlebotomy (13.2% in PHL and 7.9% in HU, respectively, *p* = 0.006). Transformation to MF was more frequent in patients treated with phlebotomy alone [16]. The NCCN and ELN recommend HU as the first-line cytoreductive treatment for elderly patients with PV [6,56]. In MPN patients (including those with PV), HU at a dose of 20–30 mg/Kg twice or thrice weekly has been found to be very effective and well tolerated, achieving sustained responses over several years [57].

According to PV studies, HU’s antithrombotic effects might be due to the reduction in the number of leukocytes that contribute to thrombus formation and leukocytosis, with a greater decrease in arterial than in venous thrombosis [58]. Compared to a historical cohort that received phlebotomy alone, HU controls the disease without inducing acute leukemia and reduces thrombosis risk during early treatment [13]. A comparison of 1:2 matched PV patients treated with either phlebotomy alone or HU over a comparable treatment period revealed significant differences between phlebotomy and HU when it came to non-fatal arterial thrombotic episodes (6.3% vs. 2.4% in phlebotomy vs. HU groups, respectively, *p* = 0.002), although HU did not result in a decrease in the number of unprovoked venous thromboembolic episodes (*p* = 0.574) [59].

There was found to be a significant burden of PV-related symptoms and poor QoL among patients with PV in a real-world survey (66% high-risk and 34% low-risk) on HU across the US. In this study, patients who had discontinued HU experienced a greater disease burden and poor QoL [60]. In addition, PV was found to increase tissue factor expression in polymorphonuclear leukocytes in the absence of in vitro challenges, and this expression decreased after HU treatment [61]. In a large population-based cohort study of older adults with PV, phlebotomy and HU were associated with improved overall survival and a decreased risk of thrombosis [53]. No additional benefits of HU have been demonstrated in patients who do not have high-risk characteristics and have a well-controlled HCT [62]. While hydroxyurea and aspirin are used to treat ET, neither drug has been shown to prevent venous or arterial thrombosis [63].

There is enough evidence to suggest that HU does not cause leukemia [64]. Squamous cell cancer is estimated to occur in 3–4% of patients. There is also an increased risk of oral, genital, and lower extremity ulcers with HU [65]. Non-carcinogenic cutaneous toxicities include dystrophy of the skin and nails, dryness, violaceous papules, and dermatomyositis. Mild gastrointestinal toxicity and mucositis and transient abnormalities of renal function have been consistently found [66]. Discontinuation of HU has a frequency of approximately 16%. Furthermore, resistance to HU has been reported in 11% and 13% of patients [67].

## 5. Peginterferon

It has been suggested that interferon should be used to treat PV because it has a disease-modifying effect. In a single-center study, 470 PV patients treated for at least 12 months with interferon, HU, or phlebotomy were followed for 20 years. After 20 years, the percentage of low-risk patients who lived without myelofibrosis was 84%, 65%, and 55%, respectively (*p* < 0.001) [68]. Although interferon is beneficial for patients with low-risk polycythemia vera, it may cause debilitating side effects. This includes fatiguability, mood changes, depression, irritability, musculoskeletal pain, alopecia, confusion in elderly patients, liver toxicity/elevated liver enzymes, cytopenia, autoimmune diseases (thyroiditis), arthralgias, neuropathy, hypothyroidism, and skin rashes, along with pulmonary, cardiac, renal, or neurologic dysfunction. The dropout rate is about 20% [69].

Peginterferon and HU were compared in a randomized frontline trial in PV. The HU group initially experienced fatigue, early satiety, itching, bone pain, and fever, but many symptoms returned to baseline after 6 months. The QoL did not differ between peginterferon and hydroxyurea (MPN-SAF single item: HU *p* = 0.009, peginterferon *p* = 0.003) [70]. This randomized trial indicates superior EFS for peginterferon over HU—approximately 95% versus 84% EFS, *p* = 0.04 [71]. Peginterferon has not been shown to improve survival or quality of life in low-risk PV patients. However, other studies have shown that peginterferon improves myelofibrosis-free survival and overall response, while also normalizing marrow morphology [72].

Increasing evidence suggests that peginterferon has the potential to change the natural history of PV with deep (and sometimes durable) molecular remissions in a subset of patients, which may translate into an improved myelofibrosis-free survival and overall survival [73]. A study found that peginterferon combined with phlebotomy may benefit young patients with low-risk PV because progression to MV is a major cause of late morbidity and mortality in low-risk PV [74]. With the use of peginterferon, there is potential for treatment discontinuation if the JAK2V617F variable allele frequency (VAF) is <10%; if there is sustained complete hematological remission for ≥2 years with no thromboembolic events; or there is worsening of disease-related signs or symptoms over the entire treatment period [75]. However, this needs to be followed via regular surveillance through a JAK2 assay.

## 6. Ruxolitinib

The JAK2V617F mutation contributes to the prothrombotic state of MPN by increasing inflammatory cytokines such as IL-6 and pentraxin-3 through the overexpression of JAK/STAT signaling [76]. Cytokines play a major role in low-risk PV patients’ constitutional symptoms. Elevated levels of IL-1α, CCL4, TNFα, and IL-6 are linked to increases in spleen size, bone pain, and fatigue. In addition, they are linked to fibrosis formation and the transformation of the disease from low-risk PV to myelofibrosis or AML [77]. In PV, reducing circulating cytokines can improve constitutional symptoms [78].

In patients with PV and splenomegaly who were phlebotomy-dependent, prior treatment with HU demonstrated superior HCT control (60% vs. 20%) and complete hematological response (24% vs. 9%; *p* = 0.003) compared to the standard of care [50]. Among PV patients, HCT control was achieved in 62% vs. 19% of patients treated with ruxolitinib and the standard of care, respectively (odds ratio, 7.28; 95% CI, 3.43–15.45; *p* < 0.0001 [79].

A prospective study compared ruxolitinib to the best available therapy for newly diagnosed PV patients with resistance/intolerance to HU or phlebotomy dependency, and these patients experienced a significant reduction in their symptom burden with ruxolitinib [80]. The RESPONSE trial included patients with resistance/intolerance to HU and phlebotomy dependency, defined as two or more phlebotomies within 24 weeks prior to screening and at least one within 16 weeks before screening, The thromboembolic event rate per 100 patient years was 1.8 with randomized ruxolitinib treatment vs. 8.2 with the best available therapy [81].

In an observational study, in 1334 patients with PV who had a symptom burden relating to known HU use, known phlebotomy, or splenomegaly, ruxolitinib was found to be efficient in controlling the above-mentioned endpoints at follow-up [80]. However, treatment with ruxolitinib is not recommend in PV unless there is the presence of severe and protracted pruritus or marked splenomegaly that is not responding to other drugs. To date, no drug therapy has been shown to improve survival or prevent leukemic/fibrotic transformation in either ET or PV; therefore, treatment is primarily directed at preventing thrombotic complications [82].

## 7. Erythrocytapheresis: Indications and Practical Considerations

Erythrocytapheresis is performed using an apheresis machine to achieve cytoreduction by decreasing red cell mass as an emergency measure. This procedure can be lifesaving in certain critical situations, such as cerebrovascular accidents or acute coronary events. It is also valuable for patients requiring emergent surgical intervention. Erythrocytapheresis prolongs the interval between phlebotomy sessions and may be useful in patients who are reluctant to undergo regular phlebotomy to maintain the HCT at <0.45. However, this procedure is expensive, can incur complications including poor vascular access and hypocalcemia, and requires referral to a tertiary center [7]. Erythrocytapheresis may have several practical implications, as 25% of PV patients find phlebotomy to have a negative impact on their QoL and up to 8% of patients discontinue phlebotomy altogether as they feel worse after each session [51]. Phlebotomy also has a negative impact on performance and productivity.

## 8. Investigational Agents

In an ongoing phase 2 trial, rusfertide, a hepcidin mimetic, reduced the HCT to <45% without the need for phlebotomy in all 16 patients who were phlebotomy-naive and who received treatment [83]. In a phase 2 study (NCT03287245) of the murine double minute (MDM2) inhibitor idasanutlin that enrolled 27 patients with PV who were phlebotomy-dependent and hydroxyurea-resistant/intolerant, 56% and 50% achieved hematocrit control and complete hematological remission, respectively [84]. The studies with idasanutlin demonstrated a striking ability to target the underlying clone, with an impressive reduction in the *JAK2V617F* allele burden, suggesting an impressive ability to control HCT, target the underlying malignant clone, and reduce the need for phlebotomy [85]. Emerging data suggest that treatment with low doses of nutlin-3 (a small-molecule antagonist of MDM2 capable of promoting PV CD34+ cell apoptosis) combined with Peg IFN-α 2a can target PV hematopoietic progenitor cells, eliminating malignant hematopoietic progenitor cells [86]. Gandotinib (LY2784544), a potent inhibitor of JAK2 activity, shows increased potency for the JAK2V6l7F mutation. Gandotinib was generally well tolerated in the enrolled MPN patients, including patients with PV, and the recommended phase 2 dose of 120 mg daily was associated with clinical improvements. Hematologic response rates across these trials were greater than 50% in those with PV. This study is ongoing and results are awaited [87]. A 4-year follow up study evaluated PV patients from the three initial trials that demonstrated an overall response rate of greater than 80% with givinostat, a histone-deacetylase inhibitor [88].

## 9. Discussion

The QoL should be a critical consideration in low-risk PV treated with phlebotomy, despite the improvement in overall survival. For patients with low-risk PV, the quality of life may be more important than overall survival because of worsening psychological and sexual symptoms. Increasing patients’ well-being by incorporating QoL indicators is the goal of modern clinical practice. Studies show that patients’ QoL improves with physician–patient interactions, treatment adherence, and outcome [89]. The Food and Drug Administration and European Medical Agency do not require physicians to submit data on QoL, and QoL does not appear to play a substantial role in drug approval [90].

Polycythemia vera has similar effects on QoL to other MPNs and advanced cancers. Patients with PV have similar QoL scores to those with advanced pancreatic cancer (EORTC QLQ-30 global QoL scores—65.7–69.7) [91]. Due to its precision and value in assessing QoL, the MPN-SAF promotes greater acceptance of patient-reported outcomes for further therapy. About 80% of patients with PV reported that the disease affected their QoL [30]. A clinical symptom model that incorporates coping and living conditions should be incorporated into assessing and understanding subjective health. Health-related QoL should be routinely assessed and has been found to be helpful by several institutions before, during, and after treatment to improve services and QoL [92].

To our knowledge, this is the first review to analyze QoL issues in patients with low-risk PV. As PV-related symptoms are aggravated by iron deficiency caused by phlebotomy, patients’ QoL is adversely affected, reinforcing the need for alternative agents. As there is no direct correlation between HCT and QoL, data suggest that QoL should be discussed with the patients by the hematologists and oncologists prior to initiating therapy [32]. Employing appropriate style and content in the communication between physicians and their patients may improve decision-making [93]. The symptoms of persistent pruritus, pica (craving for ice/flour), fatigue, cognitive impairment, dementia, frequent falls, and poor exercise tolerance persisting despite adequate treatment should be discussed upfront. In addition to the pain associated with the insertion of such a large needle into a vein to collect blood, there is also the potential for hypovolemia and transportation difficulties, including travel expenses to a phlebotomy suite and to the place of work [94].

Compared to the general population, patients with low-risk PV suffer from more fatigue and sleep difficulties than the general population, with incidence reported as high as 79%. Fatigue leads to a reduction in QoL and work productivity. The frequency of phlebotomy did not improve fatigue, which was associated with worsening QoL and decreased performance [95].

When evaluating new therapeutic strategies with the primary goal of reducing the thrombotic burden in PV, the current ELN response criteria (with clinical remission defined as a hematocrit of <45% without phlebotomy, a platelet count of ≤400 × 10^9^/L, a leukocyte count of ≤10 × 10^9^/L, and a normal spleen size) and disease-related symptoms are not informative. The ELN response in PV patients does not reduce the incidence of thrombosis or improvement in QoL. In order to rapidly evaluate interventions that prevent thrombosis in PV patients, it is necessary to develop new reliable surrogate endpoints for predicting thrombosis [96].

## 10. Conclusions

The current treatment approaches for low-risk polycythemia vera, while effective in managing hematocrit levels and preventing complications, do not always provide adequate symptom relief or enhance QoL. Reassessing PV risk assessment and treatment strategies, particularly for younger patients, could lead to better management options. Hydroxyurea and peginterferon may offer potential benefits in altering disease progression, while JAK2 inhibitors could address specific symptom control. Future advancements, including molecular testing and personalized treatment approaches, hold promise for improving disease management and patient outcomes. Ongoing research and clinical trials are crucial to validating these strategies and refining treatment paradigms for low-risk PV.

## Figures and Tables

**Table 1:** Studies comparing phlebotomy alone or phlebotomy with HU/Ropeginterferon alfa-2b or Ruxolitinib in low-risk PV.

Author	Study	No.	Treatment	Findings
Marchioli 2013 [4]	Prospective	222	Phlebotomy with HCT <0.45 vs. HCT 0.45–0.50	HCT > 0.45 increases four times cardiovascular death (*p* 0.007)
Podoltsev 2018 [53]	Retrospective	820	No phlebotomy vs. Phlebotomy +HU vs. HU	Phlebotomy + HU improves O/S (*p* < 0.01)
Barbui 2021 [52]	Prospective	127	Phlebotomy vs. Phlebotomy + ropeginterferon alfa-2b	Phlebotomy + ropeginterferon alfa-2b–effective in maintaining HCT (*p* 0.007)
Vaddi 2016 [54]	Prospective	222	Ruxolitinib vs. BAT	HCT control and ≥35% reduction in spleen size with Ruxolitinib over BAT (*p* < 0.001).

HCT—hematocrit; HU—hydroxyurea; BAT—Best Available Therapy.

## Data Availability

The original contributions presented in the study are included in the article, further inquiries can be directed to the corresponding authors.
